# Nutrients mediate caffeine inhibition of *Escherichia coli*

**DOI:** 10.1111/1758-2229.13165

**Published:** 2023-05-16

**Authors:** Megan N. McConnell, Corien Bakermans

**Affiliations:** ^1^ Division of Mathematics and Natural Sciences Penn State Altoona, Pennsylvania State University Altoona Pennsylvania USA

## Abstract

The consumption of coffee and other caffeinated drinks is increasingly popular across the globe. In the United States, 90% of adults consume at least one caffeinated beverage a day. While caffeine consumption of up to 400 mg/d is not generally associated with negative effects on human health, the impact of caffeine on the gut microbiome and individual gut microbiota remains unclear. We examined the effect of caffeine on the growth rate of *Escherichia coli*, a bacterium commonly found in the human gut, when grown aerobically or anaerobically in nutrient‐rich or minimal medium. A significant negative correlation was observed between caffeine concentration and growth rate under all conditions, suggesting that caffeine can act as an antimicrobial agent when ingested. Caffeine reduced growth rates significantly more in nutrient‐poor, but not in anoxic, conditions. Given the highly variable nutrient and oxygen conditions of the gut, these results suggest a need to further explore caffeine's inhibitory effects on the gut microbiome and its relation to human health.

## INTRODUCTION

Caffeine is the most commonly used psychoactive substance in the world. Preferred caffeine sources vary by region, with tea and soda most popular in African, Asian and Pacific countries, while coffee and soda are most popular in European, North American, Latin American and Caribbean countries (Reyes & Cornelis, 2018). The preparation method affects the amount of caffeine with brewed coffee containing 2–3.8 mM caffeine; while black tea, cold brew coffee, espresso and an energy shot contain about 1.0, 6.4, 11 and 18.5 mM caffeine, respectively (Iriondo‐DeHond et al., 2021; Mayo Clinic Staff, 2022). The consumption of coffee and high‐caffeine energy drinks, typically marketed toward students, is growing in many countries (Quadra et al., 2022). In the United States, approximately 90% of the adult population consumes caffeine with an average per capita daily intake of 211 mg (Fulgoni III et al., 2015).

Caffeine consumption of up to 400 mg/d is generally not associated with negative effects on human health; however, safe or ‘optimal’ caffeine intake remains ambiguous due to limited safety data and studies suggesting both beneficial and adverse effects (Doepker et al., 2018; Iriondo‐DeHond et al., 2021). Ingested caffeine is ‘absorbed rapidly from the gastrointestinal tract and distributed throughout body water’ (Institute of Medicine (US) Committee on Military Nutrition Research, 2001) where dose determines impact. In vitro studies on gastrointestinal tissues demonstrated that low doses of caffeine (0.1–0.3 mM) are relaxing, while high doses (>0.3 mM) cause contraction followed by relaxation (Iriondo‐DeHond et al., 2021). High doses (1–10 mM) of caffeine also inhibit slow waves, which coordinate muscle contractions, in gastrointestinal tissues (Domae et al., 2008).

While diet is a major driver of microbiome composition in the gut (Donaldson et al., 2016), the effect of caffeine remains unclear. The gut microbiome is exposed to caffeine as demonstrated in rats where about 30% of the oral dose (25 mg/kg) was taken up by the microbiome in the small intestine 2 h after administration (Mukhtar et al., 2021). Coffee has been shown to alter the composition of the gut microbiome of humans (González et al., 2020; Jaquet et al., 2009) and mice (Kim et al., 2022; Nishitsuji et al., 2018); however, coffee's complex chemical composition makes it difficult to determine the role of caffeine. In vitro studies show that caffeine can affect the growth of microorganisms either positively as a carbon and nitrogen source or negatively by interfering with DNA synthesis (Gummadi et al., 2012). Caffeine is also bacteriocidal; for example, common plant pathogens were killed at concentrations from 43 to 100 mM (Sledz et al., 2015). To date, studies of individual gut microbiota were limited to growth at one caffeine concentration or one nutrient condition or were reported without growth rates (Al‐Janabi, 2011; Dash & Gummadi, 2008; Gaul & Donegan, 2015).

The present study examined the effect of caffeine on the growth of *Escherichia coli*, commonly found in the human gut. A range of caffeine (0–20 mM) concentrations was examined that has relevance to impacts on bacteria and gastrointestinal tissues, as well as to amounts in common beverages. Given the complex nutrient, oxygen and microbiome conditions of the gastrointestinal (GI) tract (Donaldson et al., 2016; Pereira & Berry, 2017; Singhal & Shah, 2020), the effect of caffeine was assessed in different nutrient and oxygen conditions. For comparison, the growth of *Salmonella enteritidis* was also examined.

## RESULTS AND DISCUSSION

**FIGURE 1:**
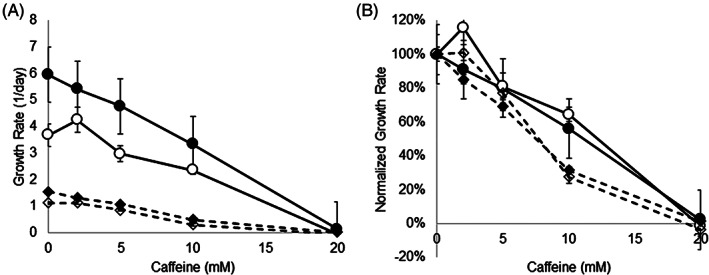
Growth rates (A) and normalized growth rates (B) of *E.coli* K12 under different caffeine, nutrient, and oxygen conditions. TSB shown with circles and solid lines, and M9 with diamonds and dashed lines. Oxic conditions shown with filled symbols, and anoxic conditions shown with unfilled symbols. Triplicate tubes were inoculated from overnight cultures and OD_600_ was monitored at 30–60‐min intervals over approximately 12 h. The growth rate (slope) and standard error of slope were calculated from the exponential phase of growth (Supplementary Data S2). Growth rates were normalized to the average rate of growth without caffeine. Separate two‐way ANOVAs were performed to assess the effect of caffeine*media and caffeine*oxygen on log‐transformed growth rates.

The growth rate of *E. coli* was significantly negatively correlated with caffeine concentration (Spearman's *r* ranged from −0.852 to −0.986, all *p* < 0.001) for all conditions (Figures 1A and S1; Table S1). For comparison, the growth rate of *S. enteritidis* was also significantly negatively correlated with caffeine concentration (Spearman's *r* ranged from −0.918 to −0.950, all *p* < 0.001) for all conditions examined (TSB and M9, oxic conditions; Figures S2 and S3). Other studies have reported similar results: the dose‐dependent inhibition of bacterial growth by caffeine has been evidenced by decreased absorbance at 18 hours (Gaul & Donegan, 2015) and increased generation times (Sledz et al., 2015). *E.coli* was more negatively impacted by caffeine than *S. enteritidis*. For instance, the growth rate of *E. coli* in TSB plus 20 mM caffeine was only 2% of its growth rate without caffeine, while the rate of *S. enteritidis* in TSB plus 20 mM caffeine was 23% of its growth rate without caffeine. Differential sensitivity of bacteria to caffeine has been described in several studies (Al‐Janabi, 2011; Gaul & Donegan, 2015; Sledz et al., 2015), where *E. coli*, *Bacillus subtilis*, *Klebsiella aerogenes*, *Salmonella typhi*, *Staphylococcus aureus* and *Enterobacter cloacae* were inhibited by 1.3, 2.6, 2.6, 2.6, 5.2 and 5.2 mM caffeine, respectively (Al‐Janabi, 2011).

Given the complex nutrient conditions of the GI tract, growth with caffeine was examined in both nutrient‐rich Trypticase Soy Broth (TSB) and nutrient‐poor M9 minimal medium (M9). A nutrient‐poor environment exacerbated the negative impact of caffeine on growth: *E. coli* was significantly more inhibited by caffeine (*F*_4,50_ = 21.9, *p* < 0.001) when grown in M9 (Figure 1B) than when grown in TSB. For example, the growth rate of *E. coli* in M9 plus 10 mM caffeine was 32% of its growth in M9 without caffeine, while the growth rate of *E. coli* in TSB plus 10 mM caffeine was 56% of its growth in TSB without caffeine. In other studies, *E.coli* was inhibited by 5.3 mM caffeine when grown in LB (Gaul & Donegan, 2015) and by 1.3 mM caffeine when grown in the more nutrient‐rich Mueller‐Hinton Broth (Al‐Janabi, 2011). However, these data are likely not directly comparable with each other or with our data because different methods were used. A nutrient‐poor environment also significantly exacerbated the negative impact of caffeine on the growth of *S. enteritidis* (*F*_4,20_ = 20.52, *p* < 0.001, Figure S3). Caffeine toxicity likely has a larger impact on bacterial growth in minimal media due to higher reproduction costs. These data indicate that nutrient‐toxin interactions should be considered, along with the composition, amount and timescale of fluctuations in nutrients (Nguyen et al., 2021), when examining the impact of diet on gut microbiome composition.

Since the partial pressure of oxygen maintained by the microbiota in the lumen is about 0.4% (Singhal & Shah, 2020), growth with caffeine under reduced oxygen conditions was examined. Notably, there was no significant difference (*F*_4,50_ = 0.070, *p* = 0.991) in the impact of caffeine on *E. coli* when grown in oxic or anoxic conditions (Figure 1B). That is, growth rates of *E. coli* decreased at the same relative amounts as caffeine concentrations increased when grown with or without oxygen, although growth was slower under anoxic conditions. To date, no comparative data has been found in the literature. These data suggest that caffeine toxicity, and mitigation thereof, is not oxygen dependent and is consistent with caffeine's dual antioxidant and pro‐oxidant properties (Azam et al., 2003).

These data demonstrate that bacterial growth inhibition by caffeine is condition dependent and will influence our understanding of how bacteria respond to caffeine in situ. This information could be particularly beneficial to special situations in human health. For example, pre‐term infants are given high doses (20 mg/kg loading dose of caffeine citrate followed by 5–10 mg/kg daily) of caffeine to stimulate the respiratory system (Rostas & McPherson, 2019) and may also benefit greatly from microbiome therapy (Henderickx et al., 2019); contraindications between the two therapies should be evaluated. Further, caffeine is an environmental contaminant of concern that may impact both free‐living and host‐associated microorganisms, given that caffeine can bioaccumulate to relevant concentrations in organisms like fish (Vieira et al., 2022).

## CONCLUSIONS

A strong negative correlation was observed between caffeine concentration and the growth rate of *E.coli* under all nutrient and oxygen conditions, suggesting that caffeine can act as an antimicrobial agent when ingested. Caffeine reduced growth rates significantly more in nutrient‐poor, but not in anoxic, conditions. Given the highly variable nutrient and oxygen conditions of the gut, these results indicate a need to further explore the extent of caffeine's inhibitory effects on the gut microbiome and its relation to human health.

## AUTHOR CONTRIBUTIONS

**Megan N. McConnell:** Conceptualization (lead); funding acquisition (equal); investigation (lead); methodology (supporting); writing – original draft (lead). **Corien Bakermans:** Conceptualization (supporting); formal analysis (lead); funding acquisition (equal); methodology (lead); supervision (lead); writing – original draft (supporting); writing – review and editing (lead).

## Supporting information


**Data S1:** Supplementary materials and methods, Supplementary Figure 1 which shows *E. coli* growth curves, Supplementary Figure 2 which shows *S. enteritidis* growth curves, Supplementary Figure 3 which shows *S. enteritidis* growth rates, and Supplementary Table 1 which provides calculated growth rates.

**Data S2:** The associated excel file contains raw data (optical density time points).

## Data Availability

All data generated or analysed during this study are included in this submitted article and its supporting information files.

## References

[emi413165-bib-0001] Al‐Janabi, A. (2011) Potential activity of the purine compounds caffeine and aminophylline on bacteria. Journal of Global Infectious Diseases, 3, 133–137. 21731299 10.4103/0974-777X.81689 PMC3125025

[emi413165-bib-0002] Azam, S. , Hadi, N. , Khan, N.U. & Hadi, S.M. (2003) Antioxidant and prooxidant properties of caffeine, theobromine and xanthine. Medical Science Monitor: International Medical Journal of Experimental and Clinical Research, 9, BR325–BR330. 12960921

[emi413165-bib-0003] Dash, S.S. & Gummadi, S.N. (2008) Inhibitory effect of caffeine on growth of various bacterial strains. Research Journal of Microbiology, 3, 457–465.

[emi413165-bib-0004] Doepker, C. , Franke, K. , Myers, E. , Goldberger, J.J. , Lieberman, H.R. , O'Brien, C. et al. (2018) key findings and implications of a recent systematic review of the potential adverse effects of caffeine consumption in healthy adults, pregnant women, adolescents, and children. Nutrients, 10, 1536. 30340340 10.3390/nu10101536 PMC6212940

[emi413165-bib-0005] Domae, K. , Hashitani, H. & Suzuki, H. (2008) Regional differences in the frequency of slow waves in smooth muscle of the Guinea‐pig stomach. Journal of Smooth Muscle Research Nihon Heikatsukin Gakkai kikanshi, 44, 231–248. 19234377 10.1540/jsmr.44.231

[emi413165-bib-0006] Donaldson, G.P. , Lee, S.M. & Mazmanian, S.K. (2016) Gut biogeography of the bacterial microbiota. Nature Reviews Microbiology, 14, 20–32. 26499895 10.1038/nrmicro3552 PMC4837114

[emi413165-bib-0007] Fulgoni, V.L., III , Keast, D.R. & Lieberman, H.R. (2015) Trends in intake and sources of caffeine in the diets of US adults: 2001–2010. The American Journal of Clinical Nutrition, 101, 1081–1087. 25832334 10.3945/ajcn.113.080077

[emi413165-bib-0008] Gaul, J. & Donegan, K. (2015) Caffeine and its effect on bacteria growth. The Journal of Biological Sciences, 1, 4–8.

[emi413165-bib-0009] González, S. , Salazar, N. , Ruiz‐Saavedra, S. , Gómez‐Martín, M. , de Los Reyes‐Gavilán, C.G. & Gueimonde, M. (2020) Long‐term coffee consumption is associated with fecal microbial composition in humans. Nutrients, 12, 1287. 32369976 10.3390/nu12051287 PMC7282261

[emi413165-bib-0010] Gummadi, S.N. , Bhavya, B. & Ashok, N. (2012) Physiology, biochemistry and possible applications of microbial caffeine degradation. Applied Microbiology and Biotechnology, 93, 545–554. 22139018 10.1007/s00253-011-3737-x

[emi413165-bib-0011] Henderickx, J.G.E. , Zwittink, R.D. , van Lingen, R.A. , Knol, J. & Belzer, C. (2019) The preterm gut microbiota: an inconspicuous challenge in nutritional neonatal care. Frontiers in Cellular and Infection Microbiology, 9, 85. 31001489 10.3389/fcimb.2019.00085 PMC6454191

[emi413165-bib-0012] Institute of Medicine (US) Committee on Military Nutrition Research . (2001) Pharmacology of caffeine. In: Caffeine for the sustainment of mental task performance: formulations for military operations. (Washington (DC): National Academies Press (US). 25057583

[emi413165-bib-0013] Iriondo‐DeHond, A. , Uranga, J.A. , Del Castillo, M.D. & Abalo, R. (2021) Effects of coffee and its components on the gastrointestinal tract and the brain‐gut axis. Nutrients, 13, 88. 10.3390/nu13010088 PMC7824117 33383958

[emi413165-bib-0014] Jaquet, M. , Rochat, I. , Moulin, J. , Cavin, C. & Bibiloni, R. (2009) Impact of coffee consumption on the gut microbiota: a human volunteer study. International Journal of Food Microbiology, 130, 117–121. 19217682 10.1016/j.ijfoodmicro.2009.01.011

[emi413165-bib-0015] Kim, J.K. , Choi, M.S. , Yoo, H.H. & Kim, D.H. (2022) The intake of coffee increases the absorption of aspirin in mice by modifying gut microbiome. Pharmaceutics, 14, 746. 35456580 10.3390/pharmaceutics14040746 PMC9031453

[emi413165-bib-0016] Mayo Clinic Staff . (2022) Caffeine content for coffee, tea, soda and more. Rochester, MN: Mayo Foundation for Medical Education and Research (MFMER).

[emi413165-bib-0017] Mukhtar, I. , Iftikhar, A. , Imran, M. , Ijaz, M.U. , Irfan, S. & Anwar, H. (2021) The competitive absorption by the gut microbiome suggests the first‐order absorption kinetics of caffeine. Dose‐Response, 19, 15593258211033111. 34421438 10.1177/15593258211033111 PMC8375357

[emi413165-bib-0018] Nguyen, J. , Fernandez, V. , Pontrelli, S. , Sauer, U. , Ackermann, M. & Stocker, R. (2021) A distinct growth physiology enhances bacterial growth under rapid nutrient fluctuations. Nature Communications, 12, 3662. 10.1038/s41467-021-23439-8 PMC8209047 34135315

[emi413165-bib-0019] Nishitsuji, K. , Watanabe, S. , Xiao, J.Z. , Nagatomo, R. , Ogawa, H. , Tsunematsu, T. et al. (2018) Effect of coffee or coffee components on gut microbiome and short‐chain fatty acids in a mouse model of metabolic syndrome. Scientific Reports, 8, 16173. 30385796 10.1038/s41598-018-34571-9 PMC6212590

[emi413165-bib-0020] Pereira, F.C. & Berry, D. (2017) Microbial nutrient niches in the gut. Environmental Microbiology, 19, 1366–1378. 28035742 10.1111/1462-2920.13659 PMC5412925

[emi413165-bib-0021] Quadra, G.R. , Brovini, E.M. , dos Santos, J.A. & Paranaíba, J.R. (2022) Caffeine consumption over time. In: Handbook of substance misuse and addictions. New York City: Springer, Cham, pp. 1–18.

[emi413165-bib-0022] Reyes, C.M. & Cornelis, M.C. (2018) Caffeine in the diet: country‐level consumption and guidelines. Nutrients, 10, 1772. 30445721 10.3390/nu10111772 PMC6266969

[emi413165-bib-0023] Rostas, S.E. & McPherson, C. (2019) Caffeine therapy in preterm infants: the dose (and timing) make the medicine. Neonatal Network: NN, 38, 365–374. 31712401 10.1891/0730-0832.38.6.365

[emi413165-bib-0024] Singhal, R. & Shah, Y.M. (2020) Oxygen battle in the gut: hypoxia and hypoxia‐inducible factors in metabolic and inflammatory responses in the intestine. The Journal of Biological Chemistry, 295, 10493–10505. 32503843 10.1074/jbc.REV120.011188 PMC7383395

[emi413165-bib-0025] Sledz, W. , Los, E. , Paczek, A. , Rischka, J. , Motyka, A. , Zoledowska, S. et al. (2015) Antibacterial activity of caffeine against plant pathogenic bacteria. Acta Biochimica Polonica, 62, 605–612. 26307771 10.18388/abp.2015_1092

[emi413165-bib-0026] Vieira, L.R. , Soares, A. & Freitas, R. (2022) Caffeine as a contaminant of concern: a review on concentrations and impacts in marine coastal systems. Chemosphere, 286, 131675. 34358890 10.1016/j.chemosphere.2021.131675

