# Supplementary material for: Nutrients mediate caffeine inhibition of Escherichia coli
Source: Environ Microbiol Rep. 2023 May 16;15(5):422–5. doi: 10.1111/1758-2229.13165 (PMC10472513; doi:10.1111/1758-2229.13165)
Supplement: Supplementary file 1 — Data S1: Supplementary materials and methods, Supplementary Figure 1 which shows E. coli growth curves, Supplementary Figure 2 which shows S. enteritidis growth curves, Supplementary Figure 3 which shows S. enteritidis growth rates, and Supplementary Table 1 which provides calculated growth rates. [file EMI4-15-422-s002.pdf]

SUPPLEMENTARY INFORMATION

Nutrients Mediate Caffeine Inhibition of *E.coli*

Megan N McConnell and Corien Bakermans

Division of Mathematics and Natural Sciences, Penn State Altoona, Pennsylvania State University, Altoona, PA, USA

Corresponding author: Corien Bakermans, cub21@psu.edu

Supplementary Materials and Methods. Bacteria (*Escherichia coli* K12 and *Salmonella enteritidis*) were grown in nutrient-rich trypticase soy broth (TSB) medium or M9 minimal medium (M9, Cold Spring Harbor Protocols 2010 doi:10.1101/pdb.rec12295) at 37°C. Growth under oxic conditions took place in a standard benchtop shaker at 300 rpm (Barnstead MaxQ 4000). Growth under anoxic conditions took place in a rigid anaerobic chamber containing an atmosphere of 95% nitrogen and 5% carbon dioxide. Oxygen levels within the chamber were maintained at <1% and continuously monitored using an O_2 sensor (Forensics Detectors FD-90A-02). Caffeine concentrations of 0, 2, 5, 10, and 20 mM were examined. For growth curves, triplicate tubes were inoculated from overnight cultures and OD_{600} was monitored at 30 to 60 minute intervals over approximately 12 hours. Growth rate (slope) and standard error of slope were calculated from the exponential phase of growth. Spearman's correlation tests were used to assess the relationship between caffeine concentration and growth rate. For comparisons between species and conditions, rates were normalized to the average rate of growth without caffeine. Separate two-way ANOVAs were performed to assess the effect of caffeine*media and caffeine*oxygen on log transformed growth rates. Raw data available as supplementary information excel file.

Supplementary Figure 1. Growth curves of *E. coli* exposed to caffeine in different nutrient (TSB or M9) and oxygen (oxic or anoxic) conditions. Caffeine was present at 0 mM (darkest gray), 2 mM, 5 mM, 10 mM, or 20 mM (lightest gray) concentrations. Average and standard deviation of triplicate cultures is shown. Note that the x-axis and y-axis are not the same scale in all graphs.

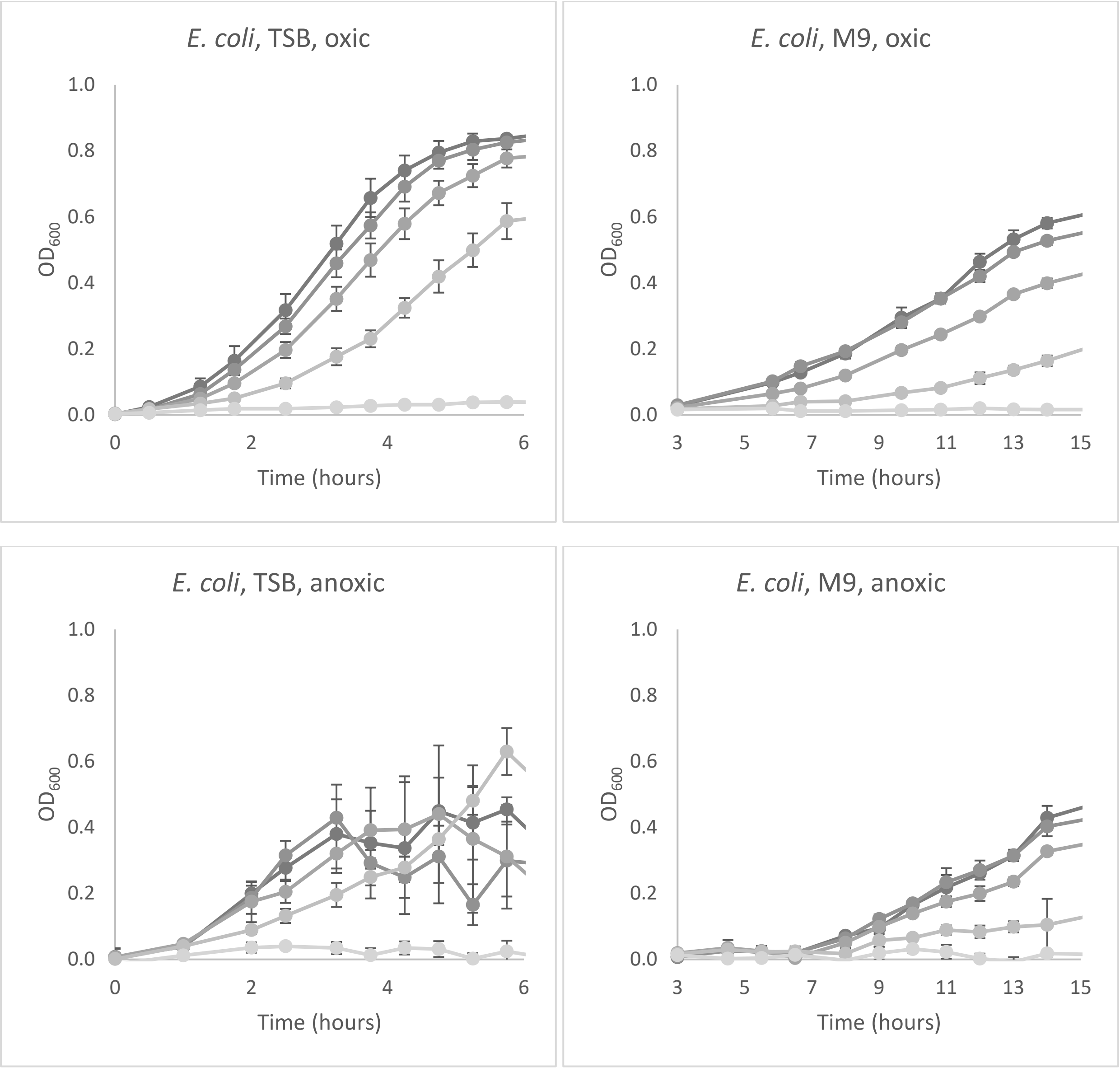

Supplementary Figure 2. Growth curves of *S. enteritidis* exposed to caffeine in different nutrient (TSB or M9) conditions. Caffeine was present at 0 mM (darkest gray), 2 mM, 5 mM, 10 mM, or 20 mM (lightest gray) concentrations. Average and standard deviation of triplicate cultures is shown.

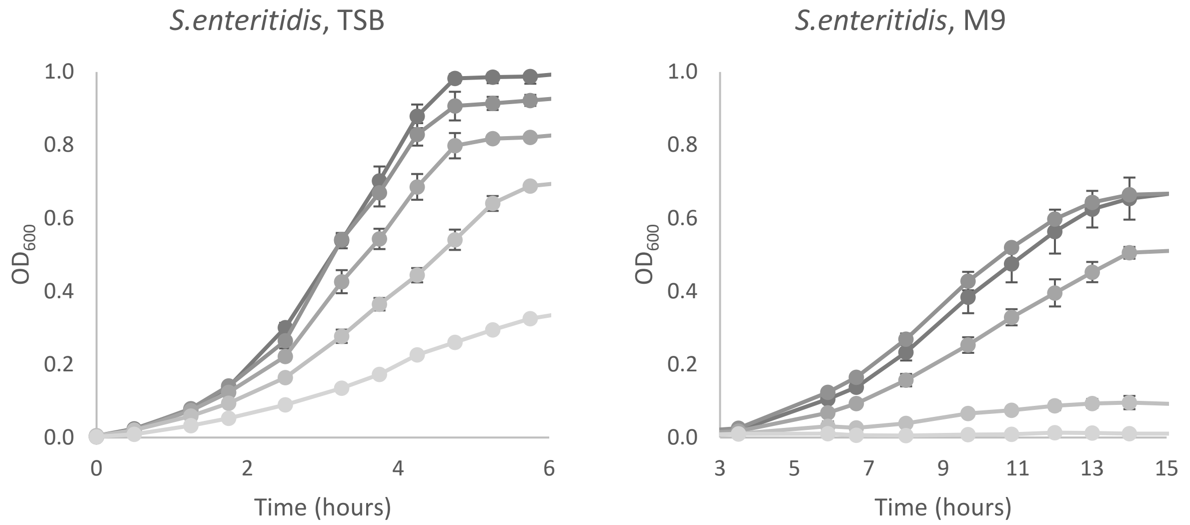

Supplementary Figure 3. Growth rates (A) and normalized growth rates (B) of *S. enteritidis* under different caffeine and nutrient conditions. TSB shown with circles and solid lines, M9 with diamonds and dashed lines.

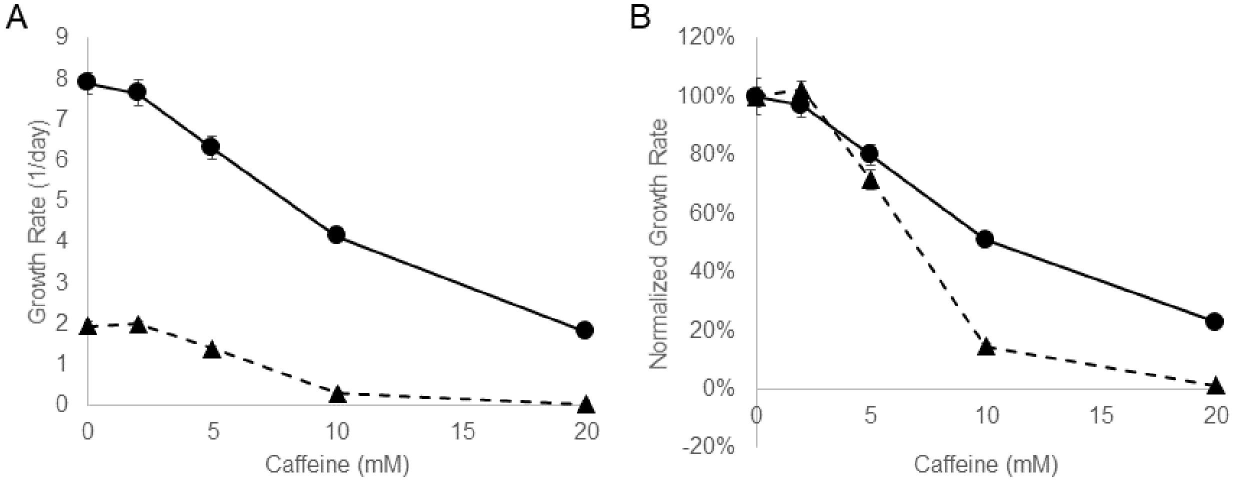

Supplementary Table 1. Growth rates of bacteria, standard error of slope reported.

	Growth rate (1/day) at different caffeine concentrations				
**Caffeine (mM)**	0	2	5	10	20
***E. coli*, TSB**	5.95 ± 0.44	5.42 ± 0.23	4.77 ± 0.20	3.33 ± 0.17	0.134 ± 0.017
***E. coli*, TSB, anoxic**	3.67 ± 0.44	4.25 ± 0.48	2.98 ± 0.29	2.35 ± 0.14	-0.048 ± 0.072
***E. coli*, M9**	6.99 ± 0.50	6.96 ± 0.26	6.30 ± 0.26	4.99 ± 0.24	2.99 ± 0.22
***E. coli*, M9, anoxic**	7.90 ± 0.28	7.66 ± 0.31	6.31 ± 0.28	4.15 ± 0.12	1.80 ± 0.04
***S. enteritidis*, TSB**	1.13 ± 0.05	1.13 ± 0.05	0.864 ± 0.033	0.312 ± 0.048	-0.048 ± 0.048
***S. enteritidis*, M9**	1.94 ± 0.12	1.99 ± 0.05	1.39 ± 0.07	0.288 ± 0.023	0.024 ± 0.008
